# The Duffy-null phenotype in Saudi Arabia: distribution, origins and clinical significance

**DOI:** 10.3389/fmed.2026.2015640

**Published:** 2026-10-28

**Authors:** Faisal Madkhali

**Affiliations:** Department of Medical Laboratory Technology, College of Nursing and Health Sciences, Jazan University, Jazan, Saudi Arabia

**Keywords:** *ACKR1*, blood transfusion, Duffy blood group system, Duffy-null associated neutrophil count, Duffy-null phenotype, Jazan, *Plasmodium vivax*, Saudi Arabia

## Abstract

**Background:**

The Duffy antigens Fy^a^ and Fy^b^, encoded by *ACKR1* (formerly *DARC*), are absent from red cells in the Fy(a−b−) phenotype, which almost always arises from homozygosity for the FY*02N.01 GATA-box variant (rs2814778). It bears on two everyday problems in non-malignant hematology, red-cell alloimmunization and the interpretation of a low neutrophil count, and confers protection against *Plasmodium vivax*. Arabian data have been phenotypic and sparse.

**Objective:**

To assemble the Saudi and Gulf evidence on Duffy-null frequency and follow it into transfusion support, neutrophil reference intervals and vivax surveillance.

**Methods:**

PubMed and Web of Science were searched from July to September 2026, pairing Duffy, *ACKR1*, *DARC* and rs2814778 with Arabian regional terms, neutropenia, reference intervals and alloimmunization. Confidence intervals are Wilson intervals recomputed from reported counts.

**Results:**

Provincial Fy(a−b−) frequency ranges from 26.7% in the north to 78.3% in Jazan, with Oman (68.5%) nearest and lower figures in the lower Gulf, consistent with enriched African ancestry at *ACKR1*. In male Jazan donors the Duffy-null neutrophil interval was 1,140–5,290/µL and institutional limits classified 50.9% as neutropenic; isolated neutropenia reaches 21% locally. Duffy alloimmunization is documented at every Saudi center examined, exclusively as anti-Fy^a^, though the rates are not directly comparable.

**Conclusion:**

Jazan carries among the highest Duffy-null frequencies reported outside sub-Saharan Africa, with direct consequences for transfusion support and for neutrophil reference intervals. One western Saudi cohort has been genotyped at the *FY* promoter; the southwest, where frequencies are highest, has not, and closing that gap is the most tractable next step.

## 1 Introduction

The Duffy antigen receptor is the product of the *ACKR1* gene on chromosome 1q23.2, an atypical chemokine receptor expressed on erythrocytes and endothelium. Two co-dominant alleles, FY*A and FY*B, differ by a single amino acid substitution (Gly42Asp) and give the Fy(a + b−), Fy(a−b+) and Fy(a + b+) phenotypes. The fourth phenotype, Fy(a−b−) or Duffy-null, arises not from coding change but from a substitution in the GATA-1 binding motif of the FY*B promoter (−67T > C; rs2814778), which abolishes erythroid expression while leaving endothelial expression intact (1). The receptor's wider roles in inflammation, hematopoiesis and tumor biology fall outside this review (2).

Duffy-null is the most distinctive red-cell polymorphism of sub-Saharan African populations, and its footprint tracks the historical range of *Plasmodium vivax*. Erythroid loss of the receptor confers resistance to *P. vivax* invasion (3), making the antigen a textbook case of malaria-driven selection. It also matters in two further everyday areas of laboratory medicine: alloantibodies to Fy^a^ and Fy^b^ complicate transfusion support, and Duffy-null individuals have constitutionally lower neutrophil counts, a benign trait that can be mistaken for pathological neutropenia.

Recent reviews have set out the clinical consequences of Duffy-null status at a global level (4, 5). Neither review assembles the primary Arabian Peninsula data, and neither addresses the region where the global model's predictions were least certain. When the global distribution of the Duffy blood group was first modeled, the Arabian Peninsula was among the regions where predictions carried greater uncertainty, since the available data were phenotypic and sparse (6). That still describes the regional evidence base, and it is the starting point here. This review assembles the Saudi and Gulf evidence, sets it against the population history that produced it, and follows it into vivax surveillance, transfusion practice and hematology reference intervals. PubMed and Web of Science searches were run between July and September 2026, combining Duffy, Fy^a^, Fy^b^, *ACKR1*, *DARC* and rs2814778 with Saudi, Jazan, Yemen, Oman, Gulf and Arab terms, and separately with neutropenia, reference interval, *Plasmodium vivax* and alloimmunization. Primary reports and reviews in English were eligible if they reported Duffy phenotype or FY genotype frequencies in Arabian Peninsula populations, or Duffy-linked neutrophil, alloimmunization or vivax data bearing on them; conference abstracts were not counted as primary evidence but are noted where no peer-reviewed figure exists. Screening and selection were carried out by the sole author, and no formal quality appraisal was undertaken, as is conventional for a narrative mini review. Where a source's own figures conflicted, the value consistent with its antigen counts was carried forward and the conflict noted in place. Wilson score 95% confidence intervals were calculated from the numerator and denominator reported by each study. Table 1 and Figure 1 were prepared in R 4.6.1 using ggplot2 4.0.3.

**Table 1:** Duffy Fy(a−b−) phenotype frequencies reported for Saudi regions and Gulf comparators.

Region/country	Study	n	Method (basis)	Fy(a−b−) % (95% CI)	Notes
Jazan, SW	Halawani 2021	143	Serology, gel — phenotype	78.3 (70.9–84.3)	Highest reported point estimate
Al-Ahsa, E	Kuriri 2024	1,549	Serology, gel — phenotype	74.1 (71.9–76.3)	
Eastern	Owaidah 2020	100	Serology, gel — phenotype	61.0 (51.2–70.0)	8-system panel
Jeddah/West	Halawani 2025	21,496	Serology, solid phase — phenotype	50.1 (49.4–50.7)	Non-Saudi donors 33.8% (*n* = 4,115)
Jeddah/Makkah (molecular)	Bawazir 2021	349	PCR + sequencing — genotype	40.7 (35.7–45.9)	FY*02N.01 homozygous; 33.5% heterozygous carriers
Al-Jouf, N	Alanazi 2026	240	Serology — phenotype	26.7 (21.5–32.6)	Lowest Saudi provincial value
Turaba, W	Mustafa 2016, cited in Halawani 2025	400	Serology — phenotype	18.0 (14.5–22.1)	Study of particular tribes
Oman	Al-Riyami 2019	337	Serology — phenotype	68.5 (63.4–73.3)	Commonest Duffy phenotype
Oman (molecular)	Al-Riyami 2022	112^a^	Molecular, FluoGene — genotype	72.3^a^ (63.4–79.8)	FY*02N.01 homozygous, 81/112
UAE (Sharjah)	Sajwani 2021	500	Serology — phenotype	11.0 (8.5–14.0)	
Kuwait	Ameen 2020	917	SNP array — predicted phenotype	2.7^b^ (1.9–4.0)	Predicted, 25/917
Arab migrants (Levant, Iraq, Gulf)	Flesch 2020	123	MALDI-TOF/PCR — genotype	3.3^c^ (1.3–8.1)	Het 8.9%; 115 of 123 from Iraq or the Levant

All Saudi studies are serological apart from Bawazir 2021, which is molecular. Phenotype rows report observed serological phenotypes; genotype and predicted-phenotype rows are not directly comparable with them. Percentages are reported to one decimal place; 95% confidence intervals are Wilson intervals computed from the reported n.

a Genotype frequency (FY*02N.01 homozygous, 81 of the 112 samples giving interpretable Duffy results, from 175 donors genotyped) rather than a serological phenotype.

b Predicted from SNP array; the same study reports GATA −67T > C in the homozygous state in 140 of 917 donors (15.3%).

c FY*02N.01 homozygous in 4 of 123 donors and heterozygous in 11 (8.9%), in the subgroup that study labels Arabian Peninsula; 115 of those donors originated in Iraq, Lebanon, Jordan, Israel or Palestine and only 8 on the Peninsula itself, so the row is a comparator for migrant Arab populations. Figures for that study's whole 1,111-donor panel were 2.0% homozygous and 16.2% heterozygous.

**Figure 1:**
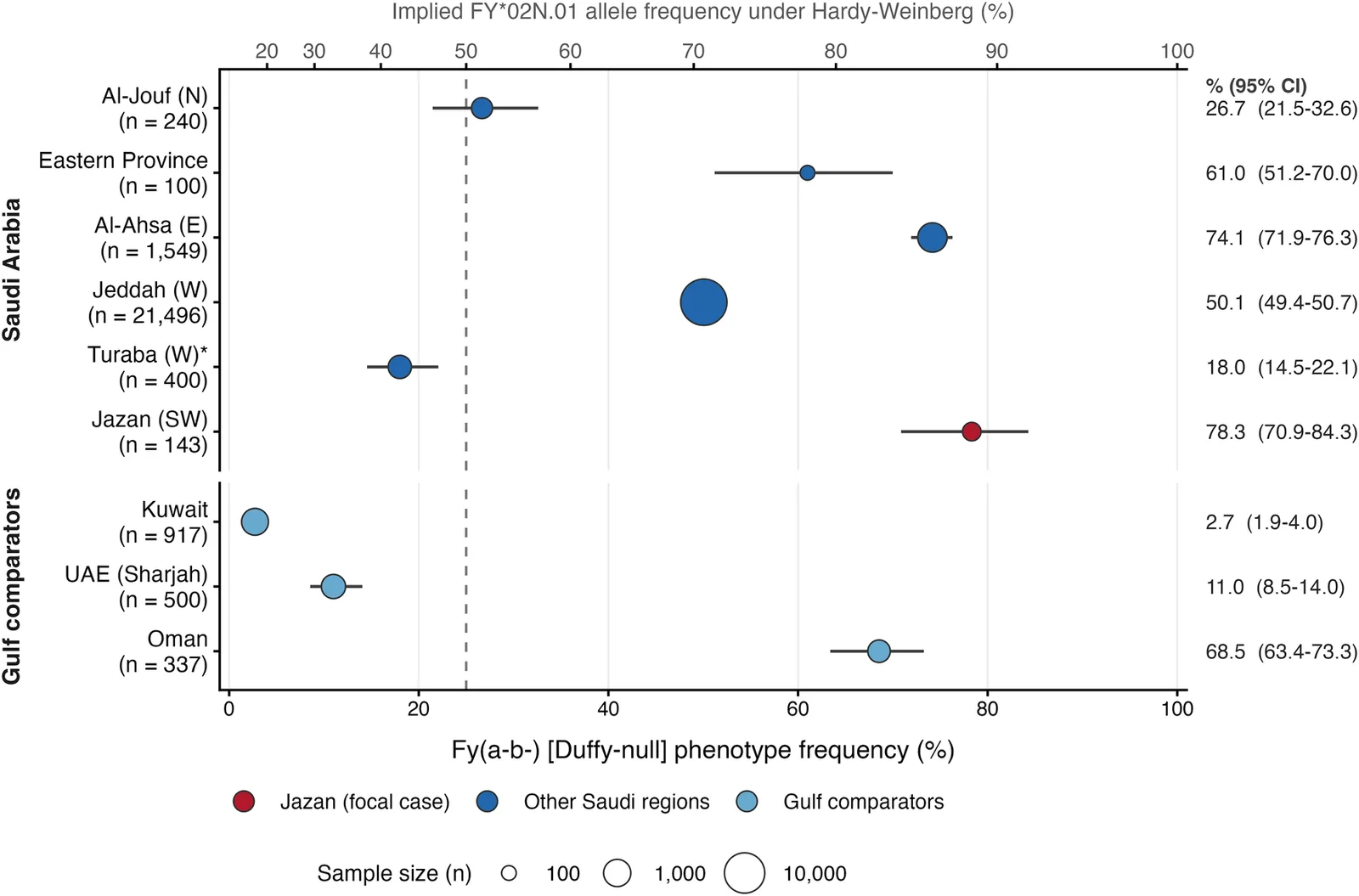
Duffy-null [Fy(a−b−)] phenotype frequency across Saudi regions (Jazan highlighted) and Gulf comparators, ordered from north to south by latitude within each panel. Points are the frequencies reported by each source, with point size increasing with sample size on a compressed scale so that small series remain visible; area is not proportional to n. Horizontal bars are 95% Wilson score confidence intervals, computed from each study's reported denominator and its reported count where given, or from the count implied by the reported percentage otherwise; each row's estimate and interval are printed at the right. Intervals describe within-study sampling error only and are not comparable across rows, which differ in method, donor selection and era. The dashed line at 25% is the Fy(a−b−) frequency implied under Hardy-Weinberg by the FY*02N.01 allele frequency of at least 50% that the global model predicted for the Peninsula (6); because that prediction is open-ended, the line is a floor rather than a benchmark. The upper axis gives, as a percentage, the allele frequency implied by each observed phenotype frequency under the same assumption. That model reports that Hardy-Weinberg was not strongly supported in its own validation, and tribal endogamy and consanguinity raise homozygote frequency above the square of the allele frequency, so the upper axis is an upper estimate rather than a measurement. The asterisk denotes a study of particular tribes rather than a provincial sample. The Al-Ahsa point and interval derive from the 1,149 of 1,549 donors the source reports, a proportion the source prints as 74.1%. The two rows labelled molecular in Table 1, and the Arab migrant row, are not plotted; the Kuwaiti value is plotted although it too is a molecular prediction rather than a serological phenotype.

## 2 Regional distribution in Saudi Arabia and the Gulf

Duffy-null is common throughout Saudi Arabia but unevenly distributed, with the highest frequencies in the southwest and the lowest in the north. In Jazan Province, Fy(a−b−) was found in 78.3% of 143 Saudi donors, the highest figure reported on the Arabian Peninsula (7). The largest dataset, 21,496 Saudi donors at King Abdulaziz Medical City in the west, gave 50.1%, significantly higher than the 33.8% recorded among non-Saudi donors at the same center, indicating that the trait belongs to the indigenous population rather than to an expatriate donor pool (8). The lowest Saudi provincial value is 26.7%, among 240 donors in Al-Qurayyat, Al-Jouf (9). Al-Ahsa (74.1%) (10) and a separate Eastern Province series (61%) (11) fall between these extremes; Al-Ahsa lies within the Eastern Province, but the two were recruited separately, so they are not one provincial estimate (Table 1; Figure 1).

Gulf comparators sit within the same envelope, and several add a molecular dimension. Omani donors show Fy(a−b−) as their commonest Duffy phenotype, at 68.5% (12), and targeted genotyping of 175 Omani donors found, in the 112 with interpretable Duffy results, the FY*02N.01 (GATA-null) allele homozygous in 81, or 72.3%, confirming the serological picture at the DNA level (13). In a multinational donor series sampled in China, 49 Yemeni donors showed Fy^a^ in 22.45% and Fy^b^ in 18.37%, implying an Fy(a−b−) frequency of at least 59.2% (29 of 49 donors); across that whole series the GATA −67T > C variant accounted for 96.8% (61/63) of nulls (14). Reported frequencies are lower further north, at 11.0% in Sharjah, UAE (15), and 2.7% in Kuwait (16). Ameen et al. also report the GATA −67T > C variant in homozygous form in 140 of 917 donors (15.3%); the two figures cannot both be right, and its own Fy^a^ (48.1%) and Fy^b^ (83.6%) frequencies imply a null phenotype near 2%, so 2.7% is the value carried into Table 1 and Figure 1. In a migrant panel genotyped in Germany, 3.3% of 123 donors were homozygous for FY*02N.01 and 8.9% were heterozygous; 115 of those 123 originated in Iraq or the Levant, however, so that figure describes migrant Arab populations rather than the Peninsula itself (Table 1) (17).

The surveys differ considerably in size, from 100 to more than 21,000 donors, and in method, spanning gel card and solid-phase serology, PCR-based genotyping, SNP array and mass spectrometry. Every Saudi and Gulf estimate in Table 1 derives from blood donors, who are health-screened, predominantly male and of working age, and are therefore not a random sample of the populations from which they are drawn; these are donor frequencies, and population frequencies may differ. The Jazan estimate, which anchors the southwestern end of the range, rests on 143 donors and would benefit from replication in a larger cohort. Jazan does not differ significantly from Al-Ahsa (78.3% against 74.1%; *p* = 0.28), so the top of the ranking is unresolved, although both exceed the Eastern Province series (*p* = 0.003 and *p* = 0.004). With 143 and 100 donors, the Jazan and Eastern Province estimates carry the widest intervals in the table, and comparisons among the smaller cohorts have limited power to detect differences of the size seen here. Overlapping confidence intervals are not in themselves evidence of no difference, and the intervals describe within-study sampling error only. The Turaba figure describes particular tribes rather than a provincial sample. The four molecular rows report genotypes or predicted phenotypes rather than serological observations, and the two are not interchangeable: serology scores an observed phenotype but cannot identify heterozygous carriers or separate the FY*02N.01 GATA null from rarer coding nulls, whereas genotype-based figures count a defined allele and predicted phenotypes are inferred rather than observed. The western region shows the distance between them, 40.7% homozygous for the GATA null by genotype (19) against 50.1% Fy(a−b−) by serology in a larger series at a different center (8). No peer-reviewed survey of the central region was identified; a conference report of 36% is the only figure available, cited in (11). Table 1 is therefore best read as a consistent regional pattern rather than as a precise cline.

Two observations follow. At approximately 78%, the Jazan value approaches the range seen in parts of sub-Saharan Africa and on the East African islands, where Fy(a−b−) reaches 86% on Grande Comore (18). Under Hardy-Weinberg it implies an FY*02N.01 (formerly FY*BES) allele frequency of about 0.89, at the upper end of, but not in conflict with, the open-ended figure above 0.50 that the global model predicted for the Peninsula; that modeled figure is an allele frequency, and its phenotype equivalent of 25% is a floor rather than a benchmark (6). Second, only one Saudi dataset is molecular: in a western cohort of 349 donors, 40.7% were homozygous for the GATA null (19), against 50.1% by serology in a larger regional series (8). Elsewhere the molecular basis has been confirmed in Oman and in a multinational panel that included Yemeni donors. Why Duffy-null is so common across the country is a question of population history.

## 3 Population history and selection at *ACKR1*

The high frequencies across Saudi Arabia are most simply explained by historical gene flow from East Africa, and genome-wide data support that explanation, although none of it resolves geography below country level. A survey of 741,000 variants in 420 Arabians and 80 Iranians found the sub-Saharan African contribution to be greatest in the west of the Peninsula, with South Asian and Levantine influence stronger in the east, and dated the main events to Islamization and the Indian Ocean slave trade (20). The same study found the African malaria-resistance signal at the *ACKR1* locus enriched in all Arabian genomes, although local African ancestry at the locus was highest in Yemen, at 0.61, and lowest of the four Arabian populations in Saudi Arabia, at 0.28, with Oman at 0.49 and the United Arab Emirates at 0.35 (20). That enrichment is evidence of a shared Peninsula-wide African legacy at this locus rather than of a gradient within Saudi Arabia, although the same study separately reports a selection signal at the locus in Saudi genomes. The Red Sea coast and the Tihama lowlands of Jazan lay directly on those routes. The distribution is not a simple west-to-east cline: Al-Ahsa in the east reaches 74.1%, above the 50.1% recorded in 21,496 western donors at Jeddah. The Turaba sample, drawn from particular tribes, falls to 18%, so tribal structure and endogamy shape local frequencies as much as longitude does, and the pattern is better described as high across Saudi Arabia and Oman but much lower in Kuwait and the UAE.

Neighboring populations show the mechanism in finer detail. In Oman, African-ancestry alleles at the Duffy locus were introduced by early admixture and then rose under positive selection: genome-wide African ancestry averaged 15.6%, but at *ACKR1* itself it reached about 76%. That study also estimated the selection coefficient at FY*02N.01 as 0.038 (95% CI 0.027–0.051), with introduction at least 59 generations ago (21). A higher value of about 0.10 has been estimated at rs2814778 in Sudanese Copts after admixture roughly 1,000–1,500 years ago, though from a small, drift-affected sample (22). The Makranis of coastal Pakistan show the same pattern, admixture followed by post-admixture selection at this locus (23).

Older serological work is consistent with this reading. In an Eastern Province series, Fy(a−b−) co-occurred with the sickle cell trait more often than chance allowed, which the authors read as evidence of African gene flow; they further proposed that AS with Fy(a−b−) should be co-selected where both *falciparum* and *vivax* circulate (24). No southwestern cohort has been examined for this co-occurrence. On present evidence, Jazan is best understood not as an anomaly but as one of the Arabian high points of a well-characterized African genetic legacy.

## 4 *Plasmodium vivax* susceptibility

The malaria pressure that shaped this distribution also makes Duffy-null status epidemiologically relevant today, through its relationship with *Plasmodium vivax*. Erythroid loss of the receptor was long held to make red cells refractory (3), which predicts the near-absence of *P. vivax* wherever the null is common. That picture has since been refined. A systematic review and meta-analysis in Sudan found that a pooled 11.7% (95% CI 7.2%–17.3%) of confirmed *P. vivax* infections occurred in Duffy-negative hosts, together with substantial diversity in the *P. vivax* Duffy Binding Protein (25). Duffy-negativity reduces susceptibility without abolishing it, an observation now documented across sub-Saharan Africa (26). Host Duffy genotype may also bear on onward transmission: mosquitoes fed on blood from FY*B/FY*02N.01 heterozygotes developed significantly higher *P. vivax* oocyst loads than on other genotypes, although no Duffy-null hosts were studied (27).

This bears directly on Jazan, which with neighboring Aseer carries what remains of malaria transmission in Saudi Arabia (28, 29). *Anopheles arabiensis*, the principal vector in southwestern Saudi Arabia, is present throughout the Tihama lowlands (29, 30). In a low-transmission Jazan cohort, PCR point prevalence was 10.9% and 80% of infections were imported. Of the 30 PCR-confirmed infections, 23 were *P. falciparum*, 5 *P. vivax* and 2 mixed, although all *P. vivax* mono-infections were in migrants and Duffy status was not determined (28). Nationally, 13,852 cases were reported between 2017 and 2021, with the southern regions at highest risk from importation across the Yemeni border, and with combined *P. vivax* and *P. ovale* cases falling from 885 in 2017 to 419 in 2020 before rising to 480 in 2021 (29). Saudi Arabia has reduced autochthonous malaria by more than 99% since the late 1990s and is close to elimination (30). Even so, continuing importation from a neighbor with uninterrupted transmission, competent vectors and a highly Duffy-null host population together keep the vivax question locally relevant. Host genetic susceptibility is already under study in the same population (31, 32).

## 5 Transfusion and alloimmunization

A population that is majority Duffy-null changes the transfusion calculus in two directions. Because most donors and patients in Jazan and the Eastern Province lack Fy^a^ and Fy^b^, antigen-negative units are abundant and Duffy matching is unusually easy to deliver here. The risk runs the other way: the Duffy-null majority, not the antigen-positive minority, can form anti-Fy^a^ on exposure to antigen-positive units. This matters most for the chronically transfused, above all patients with sickle cell disease and *β*-thalassemia, both concentrated in Jazan and the Eastern Province.

Local data bear this out. Among transfused Jazan patients, red-cell alloimmunization reached 12.98% in sickle cell disease and 13.21% in thalassemia; of 64 antibodies detected, anti-E (17.19%) and anti-K (14.06%) led, while anti-Fy^a^ accounted for two (3.13%) and anti-Fy^b^ for none, although 14 (25%) of the sickle cell antibodies were of undetermined specificity (33). A Saudi pediatric sickle cell series found alloantibodies in 17.4% of patients, led by anti-K, anti-E and anti-S, with anti-Fy^a^ in one patient (4.8%) (34), and a Jeddah series of 241 sickle cell patients detected anti-Fy^a^ in 7.5% of its alloimmunized patients, calling explicitly for phenotyping to be extended beyond Rh and Kell to include Duffy and Kidd, alongside a national donor antigen registry (35). In 1,027 Jazan patients already receiving Rh- and K-matched units, alloimmunization reached 7.6% and anti-Fyᵃ accounted for 5.6% of antibodies (36). A recent African review ranks Duffy after Rh and Kell (37), a constraint that a Duffy-null-majority donor pool relaxes. Duffy alloimmunization is therefore documented at every Saudi center examined, though the rates are not directly comparable. The antigen-negative background makes proactive matching worthwhile, provided donor and recipient antigen status is known. Antigen status is currently determined serologically, which does not identify the underlying *FY* genotype. The distinction is actionable: a patient homozygous for the GATA null still expresses Fy^b^ on non-erythroid tissue and can receive Fy^b^-positive units without concern for anti-Fy^b^, unlike the rarer coding nulls (4).

## 6 Neutrophil reference ranges

The consequence that surfaces most often in routine practice is hematological. Loss of the erythroid receptor is associated with constitutionally lower absolute neutrophil counts, now termed either Duffy-null associated neutrophil count (DANC) or *ACKR1*/*DARC*-associated neutropenia (ADAN), and previously benign ethnic neutropenia, terminology that current reviews advise against (4). Counts fall below the conventional 1,500/µL (1.5 × 10^9^/L) threshold without raising infection risk, and current reviews recommend that reference intervals accommodate it (38), anchored to Duffy status rather than to a population label (4, 39). The polymorphism is documented in an Israeli Arab pedigree study (40), and among 115 children investigated for isolated neutropenia 49 had ADAN, 29 of them in the severe range below 500/µL, yet infections were no more frequent over a median 2.5 years; the authors recommend testing any child with isolated neutropenia and no severe infection (41).

Southwestern Saudi Arabia has a substantial local evidence base. A multinational study has established Duffy-null reference intervals across four countries, including a cohort of blood donors from Jazan Province, where the Duffy-null absolute neutrophil count interval was 1,140–5,290/µL and institutional intervals, whose lower limit in Jazan is 2,500/µL, classified 50.9% of the Saudi participants as below normal (39). That donor cohort was almost entirely male, so the interval describes adult male donors and should not be extended to women or children without local validation. An independent US cohort of 122 Duffy-null adults gave a closely comparable lower limit of 1,010/µL (42). In the Jazan cohort, Duffy status and neutrophil count were measured in the same individuals, which links the two literatures directly; the high prevalence of isolated neutropenia across seven southern and western Saudi cities, 11%–23% of 91,880 complete blood counts and 21% in Jazan itself, is consistent with the same explanation, although that study measured no Duffy status (43).

Recording DANC as pathological neutropenia can lead to unnecessary referral and investigation, and it affects drug-safety monitoring, since agents such as clozapine are governed by neutrophil thresholds derived largely in European-ancestry populations (44, 45). A UK pharmacogenomics consortium has now published a guideline recommending pre-emptive *ACKR1* genotyping for people starting clozapine, with revised monitoring thresholds and a supporting health-economic case, although the test is not yet routinely offered in the National Health Service (46). For Jazan, a locally validated lower limit is a patient-safety measure rather than a refinement.

## 7 Gaps and a research agenda

The central gap is specific. Despite the six Saudi serological surveys in Table 1, the *FY* GATA promoter has been genotyped in only one Saudi cohort, in the west (19). The Jazan donor cohort described above was characterized serologically (39). The ∼78% Jazan figure therefore rests on phenotype. This matters practically: serology does not distinguish the FY*02N.01 GATA-null from rarer coding nulls, does not identify heterozygous carriers, and cannot support genotype-based transfusion matching. A recent scoping review noted the same international absence of studies applying Duffy genotype to clinical decision thresholds (5).

Closing the gap is tractable and may be cheaper than it appears. Groups in the province have genotyped Diego-system Wright antigens (47) and characterized RHD and RHCE variation in 354 Saudi and 110 non-Saudi local donors (48), and case-control malaria cohorts are already running host-genetics studies in the same population (31, 32). A second Saudi group has genotyped the coding variant (49). The Rh work is the more consequential precedent, because it shows multiplex red-cell genotyping already running in a Jazan blood bank, on the ID CORE XT platform (48). Extending it to the *FY* promoter in donor and transfused patient series would:

- quantify carrier frequency in the southwest, already 33.5% in the west (19);
- anchor a neutrophil reference range to genotype rather than phenotype; and
- give a genotype-anchored denominator for vivax surveillance, where host and parasite genotyping are now recommended together.

This review is narrative rather than systematic and depends on primary surveys that differ in size and method. All frequency estimates derive from donor cohorts rather than population samples, and serological and molecular estimates are not directly comparable. No southwestern Saudi study reporting *FY* promoter genotypes was identified, so the regional picture rests on serology and the Jazan anchor on 143 donors.

## 8 Conclusion

Saudi Arabia, and Jazan in particular, carries one of the highest Duffy-null frequencies reported outside sub-Saharan Africa. The most plausible explanation is a legacy of East African admixture along the Red Sea coast, retained by post-admixture selection, usually attributed to malaria, and genome-wide data show the African signal at *ACKR1* enriched across Arabian populations. That single polymorphism reaches into three areas of local practice. It keeps the vivax question relevant in a country approaching malaria elimination. It shapes transfusion and alloimmunization strategy for a large sickle cell and thalassemia population. And it supports Duffy-specific neutrophil reference ranges, so that a benign trait is not recorded as disease, though the local interval so far rests on male donors. The remaining gap is molecular: the southwest has only serology. Genotyping the *FY* promoter in Jazan, on a platform already in routine use there, is the obvious and achievable next step.
